# Comparison of volumetric‐modulated arc therapy and dynamic conformal arc treatment planning for cranial stereotactic radiosurgery

**DOI:** 10.1120/jacmp.v17i1.5677

**Published:** 2016-01-08

**Authors:** Jessica Molinier, Christine Kerr, Sebastien Simeon, Norbert Ailleres, Marie Charissoux, David Azria, Pascal Fenoglietto

**Affiliations:** ^1^ Service Radiothérapie, ICM Val‐d'Aurelle Montpellier France

**Keywords:** volumetric arc therapy, cranial stereotactic radiosurgery, treatment planning

## Abstract

The aim was to analyze arc therapy techniques according to the number and position of the brain lesions reported by comparing dynamic noncoplanar conformal arcs (DCA), two coplanar full arcs (${\text{RA}}_{C}$) with volumetric‐modulated arc therapy (VMAT), multiple noncoplanar partial arcs with VMAT (${\text{RA}}_{\text{NC}}$), and two full arcs with VMAT and 10° table rotation (${\text{RA}}_{T}$). Patients with a single lesion (n = 10), multiple lesions (n = 10) or a single lesion close to organs at risk (n = 5) and previously treated with DCA were selected. For each patient, the DCA treatment was replanned with all VMAT techniques. All DCA plans were compared with VMAT plans and evaluated in regard to the different quality indices and dosimetric parameters. For single lesion, homogeneity index (HI) better results were found for the ${\text{RA}}_{\text{NC}}$ technique (0.17±0.05) compared with DCA procedure (0.27±0.05). Concerning conformity index (CI), the ${\text{RA}}_{T}$ technique gave higher and better values (0.85±0.04) compared with those obtained with the DCA technique (0.77±0.05). DCA improved healthy brain protection (8.35±5.61 cc vs. 10.52±6.40 cc for ${\text{RA}}_{\text{NC}}$) and reduced monitor unit numbers (3046±374 MU vs. 4651±736 for ${\text{RA}}_{\text{NC}}$), even if global room occupation was higher. For multiple lesions, VMAT techniques provided better HI (0.16) than DCA (0.24±0.07). The CI was improved with ${\text{RA}}_{T}$ (0.8±0.08 for ${\text{RA}}_{T}$ vs. 0.71±0.08 for DCA). The $V_{10\text{Gy}}$ healthy brain was better protected with DCA (9.27±4.57 cc). Regarding the MU numbers: ${\text{RA}}_{\text{NC}}<{\text{RA}}_{T}<{\text{RA}}_{C}<\text{DCA}$. For a single lesion close to OAR, ${\text{RA}}_{T}$ achieved high degrees of homogeneity (0.27±0.03 vs. 0.53±0.2 for DCA) and conformity (0.72±0.06 vs. 0.56±0.13 for DCA) while sparing organs at risk ($D_{\text{max}}=12.36\pm 1.05\;\text{Gy}$ vs. 14.12±0.59 Gy for DCA, and $D_{\text{mean}}=3.96\pm 3.57\;\text{Gy}$ vs. 14.72±3.28 Gy for DCA). On the other hand, MU numbers were lower with DCA (2254±190 MU vs. 3438±457 MU for ${\text{RA}}_{\text{NC}}$) even if overall time was inferior with ${\text{RA}}_{C}$. For a single lesion, DCA provide better plan considering low doses to healthy brain even if quality indexes are better for the others techniques. For multiple lesions, ${\text{RA}}_{\text{NC}}$ seems to be the best compromise, due to the ability to deliver a good conformity and homogeneity plan while sparing healthy brain tissue. For a single lesion close to organs at risk, ${\text{RA}}_{T}$ is the most appropriate technique.

PACS numbers: 87.55. dk, 87.56.bd

## I. INTRODUCTION

Stereotactic radiosurgery consists in delivering a single high dose on a small target while sparing the surrounding healthy tissue. Consequently, this treatment requires a rigorous precision in immobilization and patient positioning. Cranial stereotactic radiosurgery was initially performed with a stereotactic invasive head frame, such as the Leksell frame.^(1)^ Several relocatable stereotactic frames, like the Gill‐Thomas‐Cosman frame,^(2)^ were proposed to replace the frame‐based system, but the setup accuracy was not convincing.^3^, ^4^, ^5^

The recent advances in imaging development allowed assessing the feasibility of frameless radiosurgery. Two image‐guidance systems are now available: the ExacTrac (ET) system from Brainlab and On‐Board Imager (OBI) system from Varian. Coupled with a robotic couch, ET allows a six‐dimensional patient positioning before and during irradiation from two floor‐mounted kV X‐ray tubes. The OBI system generates either two orthogonal planar images or a three‐dimensional image with the use of cone‐beam computed tomography (CBCT). Several studies compared these two systems and concluded they had similar accuracy.^6^, ^7^ These advances considerably increased the setup precision, and allowed the use of different types of frameless masks with a comparable accuracy to that of the invasive frame approach.^8^, ^9^, ^10^, ^11^, ^12^, ^13^

Besides a high level of precision for positioning, stereotactic radiosurgery requires a sharp dose falloff to spare the neighboring tissue. Many modalities are now capable of performing these radiosurgery treatments. With ${\;}^{60}\text{Co}$ sources, GammaKnife created collimated convergent beams to the target.^14^, ^15^ CyberKnife, a linear accelerator coupled with a 6D robotic arm, is shown to deliver multiple nonisocentric pencil beams.^(16)^ The linear accelerator (linac) also belongs to this category.^(17)^ The main techniques used with linac are the dynamic conformal arcs,^18^, ^19^ the fixed‐field, intensity‐modulated radiation therapy (IMRT)^20^, ^21^ and, more recently, the volumetric‐modulated arc therapy (VMAT).^22^, ^23^, ^24^

In this study, four rotational techniques for cranial stereotactic radiosurgery were compared: the use of two coplanar full arcs with VMAT (${\text{RA}}_{C}$) and of multiple noncoplanar partial arcs with VMAT (${\text{RA}}_{\text{NC}}$), and the two full arcs with VMAT and the 10° table rotation (${\text{RA}}_{T}$) techniques. The aim of the study was to analyze the dosimetric parameters of different arc therapy techniques according to the number and the position of the brain lesions reported.

## II. MATERIALS AND METHODS

### A. Patients

Twenty‐five patients previously treated with DCA were selected for this study leading to 100 dosimetric plans. Ten patients were irradiated for a single lesion, 10 for multiple lesions, and 5 for single lesion close to organs at risk. The diagnoses were mainly lung and breast metastasis (respectively, 32% and 16% of patients). The median PTV was 2.4 cm^3^ (range 0.68–13.7 cm^3^). The mean prescribed dose was 21 Gy (range 14–25 Gy). The mean normalization isodose (isodose covering 99% of the PTV) was 81.2%. In the case of multiple lesions, patients with the same prescribed dose to each target were selected. Patient and lesion characteristics are summarized in Table 1.

**Table 1:** Patient characteristics.

*Patient*	*Diagnosis*	*PTV Volume (cc)*	*Prescribed Dose (Gy)*	*Normalization Isodose (%)*	*No. of Arcs*
*Single Lesion*					
1	glioma	3.86	21	84	4
2	1 ovary metastasis	3.51	22.5	88.1	4
3	glioma	2.15	25	81.4	4
4	1 breast metastasis	13.7	20	79.2	4
5	1 ovary metastasis	13.3	22.5	84.4	4
6	1 melanoma metastasis	1.64	25	82.9	3
7	1 breast metastasis	2.6	22.5	87.5	3
8	1 lung metastasis	2.4	22.5	83.5	3
9	1 lung metastasis	1.5	22.5	82.5	4
10	1 lung metastasis	7.3	20	82	4
*Multiple Lesions*					
11	2 pancreas metastasis	0.69+2.68	22.5	70	3+4
12	4 breast metastasis	0.5+3.17+2.78+0.46	22.5	92.4	4+3+4+4
13	2 lung metastasis	1.91+0.57	25	5+4	
14	2 melanoma metastasis	0.79+0.56	25	81.8	4+4
15	2 lung metastasis	2.78+1.79	22.5	86.3	4+4
16	2 melanoma metastasis	1.2+2.6	22.5	82	3+3
17	2 breast metastasis	0.9+1.5	22.5	82.4	4+4
18	2 lung metastasis	1.69+5	22.5	83.6	4+3
19	2 lung metastasis	4.3+5.1	22.5	84.6	4+4
20	2 lung metastasis	5.3+2.4	20	81.5	4+3
*Single Lesion Close to OAR*					
21	Neurinoma	4.81	14	70.5	3
22	AVM	2.87	14	61.7	3
23	Meningeoma	2.17	15	72.9	3
24	Neurinoma	7.2	14	80.5	3
25	Schwannoma	1.1	15	74.7	3

### B. Cranial stereotactic radiosurgery procedure

Each patient was imaged by computed tomography (CT) (GE Healthcare, Waukesha, WI) using 1.25 mm spacing and by a T1‐weighted magnetic resonance (MR) (Siemens Heathcare, Erlangen, Germany) scan using 1 mm spacing. The images were imported into iPlan RT Image (v.4.1; Brainlab AG Feldkirchen, Germany). A neurosurgeon outlined the gross tumor volume (GTV) and the organs at risk on the registered scans. A 2 mm margin was added to GTV to create the planning target volume (PTV), and the healthy brain was defined as the total brain volume excluding PTV.

Each treatment plan was produced using iPlan RT Dose (v.4.5; Brainlab) and several dynamic noncoplanar conformal arcs (3–4 per lesion). Couch rotations varied according to the location of the lesion and were generally spaced out 40°. The gantry rotations were ranged from 330° to 210° and from 30° to 150°. Dose distributions were calculated with the pencil beam convolution (PBC) algorithm. The treatment plan was validated by physicians with regard to different criteria: the PTV coverage by 80% isodose, the healthy brain tissue volume receiving 10 Gy (<12.5 cc in our department per the Minniti et al. study^(25)^), and doses to OAR in some cases.

Finally, treatments were delivered on a Novalis TrueBeam STx linear accelerator (Varian Medical Systems, Helsinki, Finland) equipped with the Varian High Definition 120 MLC, beam energy of 6 MV, and 600 MU/min dose rate.

### C. VMAT treatment planning

**Figure 1:**
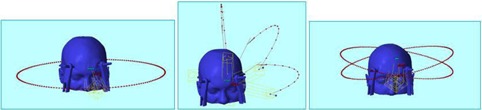
Examples of two coplanar full arcs (left), noncoplanar partial arcs (center), and two full arcs with table rotations (right).

For each patient, three additional VMAT plans were created: two coplanar full arcs (${\text{RA}}_{C}$), multiple noncoplanar partial arcs (${\text{RA}}_{\text{NC}}$), and two noncoplanar full arcs with 10° table rotations (${\text{RA}}_{T}$). These modalities are shown in Fig. 1.

#### C.1 ${\text{RA}}_{C}$

The technique consisted in two full arcs with a gantry rotation from a starting angle of 179° and stopped at 181° in the counterclockwise direction and reverse for the second arc. The collimator rotations were 45° and 315°, and the couch angle was set at 0°. The isocenter coordinates were similar to the DCA in the case of a single lesion, and the Eclipse automated isocenter was used for multiple lesions.

#### C.2 ${\text{RA}}_{\text{NC}}$

For single lesions, the beam arrangement was the same as for the DCA (3–4 arcs per lesion). In case of multiple lesions, four noncoplanar arcs were used with a single isocenter, compared with a multiple isocenter treatment for DCA. They were similar for every case of multiple lesions. The two first arcs started at 30° and stopped at 150° with 45° and 315° collimator angles and 70° and 30° couch rotations, respectively. The two others were ranged from 330° to 210°, with 0° and 90° collimator angles and 330° and 290° couch rotations, respectively. This beam arrangement allowed covering the whole brain with a single isocenter. The isocenter was placed using the Eclipse automated isocenter tool.

#### C.3 ${\text{RA}}_{T}$

The beam configuration (fields and isocenter) was identical to that of the ${\text{RA}}_{C}$, but 10° and 350° couch rotations were added for the two arcs.

The VMAT plans were generated using Eclipse treatment planning (v10, Varian Medical Systems, Helsinki, Finland) and planned on the same CT scans. They were designed using a progressive resolution algorithm (PRO, v10.0.28, Varian Medical Systems), and calculated using the anisotropic analytical algorithm (AAA, v10.0.28; Varian Medical Systems). All VMAT plans were optimized from results obtained with DCA plans. Thus, $V_{10\text{Gy}}$ of healthy brain and $D_{\text{max}}$ and $D_{\text{mean}}$ for OAR (if necessary) of the DCA plan were used as optimization objectives for VMAT plans. For the study, treatment plans of each patient were compared with the same PTV coverage. Consequently, for each patient, VMAT plans were optimized to deliver the same peripheral dose as for the DCA.

### D. Plan evaluation

DCA treatment plans are the benchmark in our department. They were compared with each VMAT plan. All of them were evaluated using quality indexes for PTVs and the dose volume histograms (DVH) for healthy brain and OAR.

The target coverage was compared with conformity and homogeneity indexes (CI and HI). The conformity index chosen was the Paddick CI^(26)^ defined as:

(1) $$CI=\frac{{\left(PTV\;\text{covered}\;by\;normalization\;isodose\right)}^{2}}{V_{PTV}*V_{normalization\;isodose}}$$

The normalization isodose was defined as the isodose covering 99% of the PTV. This index estimated both the PTV coverage and the surrounding tissue irradiation. CI was 1 when the PTV was covered by the normalization isodose while sparing the whole healthy tissue.

The HI was defined as the ratio of the difference between the maximum and minimum doses to the mean dose for the PTV:

(2) $$HI=\frac{D_{\text{max}}-D_{\text{min}}}{D_{mean}}$$

The HI assessed the dose distribution homogeneity within the PTV. The more the dose distribution was homogenous in the target the more the index reached 0.

Further, $V_{10\text{Gy}}$ for healthy brain tissue was recorded for all cases except for the case of a single lesion close to OAR. In this particular case, prescribed dose was 14 Gy and 10 Gy isodose was generally target coverage isodose. Consequently, 10 Gy isodose was not representative of the dose received by normal brain. In addition, for single lesion close to OAR, maximum and mean doses($D_{\text{max}}$ and $D_{\text{mean}}$) of the OAR were compared.

Finally, the number of monitor units (MUs) was assessed for each technique.

## III. RESULTS

The quality indexes and the MUs results for the three cases (single lesion, multiple lesions, and single lesion close to OAR) are summarized in 2, 3, 4, respectively. Doses to healthy brain (cases 1 and 2) and OAR (case 3) are presented in 2, 3, 4.

**Table 2:** Comparison of CI, HI, and MU for DCA, ${\text{RA}}_{C},\;{\text{RA}}_{\text{NC}}$, and ${\text{RA}}_{T}$ in single lesion case.

	*CI*				*HI*				*MU*			
*Patient*	*DCA*		${\text{RA}}_{\text{NC}}$	${\text{RA}}_{T}$	*DCA*	${\text{RA}}_{C}$	${\text{RA}}_{\text{NC}}$	${\text{RA}}_{T}$	*DCA*	${\text{RA}}_{C}$	${\text{RA}}_{\text{NC}}$	${\text{RA}}_{T}$
1	0.74	0.82	0.87	0.87	0.25	0.21	0.18	0.19	2876	5521	4694	5804
2	0.67	0.84	0.86	0.85	0.17	0.17	0.16	0.20	3173	3848	5131	6362
3	0.72	0.73	0.76	0.78	0.30	0.26	0.17	0.19	3693	6193	5272	5636
4	0.84	0.90	0.91	0.91	0.36	0.24	0.14	0.23	2645	3947	3916	4517
5	0.82	0.85	0.85	0.87	0.27	0.25	0.23	0.27	3071	3357	4182	3930
6	0.80	0.82	0.80	0.82	0.28	0.22	0.27	0.24	2851	7468	5812	7518
7	0.79	0.86	0.84	0.87	0.21	0.16	0.15	0.13	2686	5994	3911	5371
8	0.80	0.87	0.85	0.87	0.27	0.16	0.13	0.16	2673	5153	3560	5236
9	0.73	0.77	0.81	0.81	0.28	0.21	0.14	0.20	3599	6341	5283	6396
10	0.79	0.89	0.86	0.89	0.29	0.22	0.12	0.17	3193	5197	4746	5204
Mean	0.77	0.84	0.84	0.85	0.27	0.21	0.17	0.20	3046	5302	4651	5597
SD	0.05	0.05	0.04	0.04	0.05	0.04	0.05	0.04	374	1285	736	1014

**Table 3:** Comparison of CI, HI, and MU for DCA, ${\text{RA}}_{C},\;{\text{RA}}_{\text{NC}}$, and ${\text{RA}}_{T}$ in multiple lesions case.

	*CI*				*HI*				*MU*			
*Patient*	*DCA*	${\text{RA}}_{C}$	${\text{RA}}_{\text{NC}}$	${\text{RA}}_{T}$	*DCA*	${\text{RA}}_{C}$	${\text{RA}}_{\text{NC}}$	${\text{RA}}_{T}$	*DCA*	${\text{RA}}_{C}$	${\text{RA}}_{\text{NC}}$	${\text{RA}}_{T}$
11	0.65	0.73	0.69	0.76	0.35	0.20	0.17	0.16	6166	4110	3566	4074
12	0.60	0.70	0.74	0.74	0.13	0.23	0.19	0.21	12090	4573	4251	4617
13	0.65	0.65	0.60	0.66	0.14	0.18	0.18	0.18	8104	5685	5129	5762
14	0.64	0.65	0.59	0.68	0.26	0.14	0.15	0.13	6982	4337	4203	4134
15	0.71	0.73	0.75	0.82	0.22	0.16	0.15	0.16	6199	5500	4102	5425
16	0.73	0.86	0.84	0.86	0.29	0.14	0.16	0.13	5697	6086	4895	6297
17	0.70	0.80	0.74	0.81	0.27	0.14	0.14	0.12	6715	6194	5842	6189
18	0.80	0.85	0.80	0.85	0.18	0.14	0.15	0.14	6477	6168	5065	6103
19	0.85	0.88	0.82	0.88	0.26	0.14	0.19	0.17	5597	6537	4371	6511
20	0.80	0.90	0.82	0.90	0.30	0.16	0.19	0.16	5266	6078	6043	5848
Mean	0.71	0.77	0.74	0.80	0.24	0.16	0.17	0.16	6929	5227	4746	5496
SD	0.08	0.10	0.09	0.08	0.07	0.03	0.02	0.03	1984	873	792	905

**Table 4:** Comparison of CI, HI, and MU for DCA, ${\text{RA}}_{C},\;{\text{RA}}_{\text{NC}}$, and ${\text{RA}}_{T}$ in the case of single lesion close to an OAR.

	*CI*				*HI*				*MU*			
*Patient*	*DCA*	${\text{RA}}_{C}$	${\text{RA}}_{\text{NC}}$	${\text{RA}}_{T}$	*DCA*	${\text{RA}}_{C}$	${\text{RA}}_{\text{NC}}$	${\text{RA}}_{T}$	*DCA*	${\text{RA}}_{C}$	${\text{RA}}_{\text{NC}}$	${\text{RA}}_{T}$
21	0.64	0.62	0.70	0.71	0.47	0.35	0.29	0.25	2121	3720	2841	3392
22	0.42	0.50	0.54	0.72	0.77	0.31	0.30	0.30	2080	4305	3726	3585
23	0.40	0.72	0.52	0.79	0.70	0.30	0.29	0.25	2354	3852	3689	4670
24	0.68	0.73	0.59	0.74	0.35	0.30	0.34	0.29	2180	3481	3875	3212
25	0.64	0.66	0.65	0.62	0.34	0.21	0.23	0.25	2539	4827	3061	3823
Mean	0.56	0.65	0.60	0.72	0.53	0.29	0.29	0.27	2255	4037	3438	3736
SD	0.13	0.09	0.07	0.06	0.20	0.05	0.04	0.03	190	534	457	569

**Figure 2:**
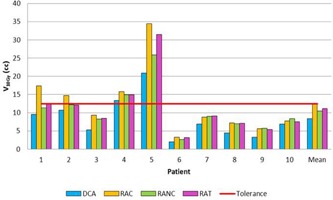
Comparison of healthy brain $V_{10\text{Gy}}$ for DCA, ${\text{RA}}_{C},\;{\text{RA}}_{\text{NC}}$, and ${\text{RA}}_{T}$ in single lesion case.

**Figure 3:**
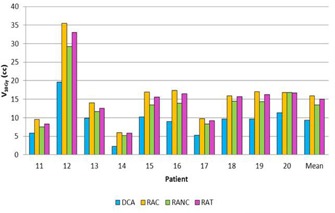
Comparison of healthy brain $V_{10\text{Gy}}$ for DCA, ${\text{RA}}_{C},\;{\text{RA}}_{\text{NC}}$, and ${\text{RA}}_{T}$ in multiple lesion case.

**Figure 4:**
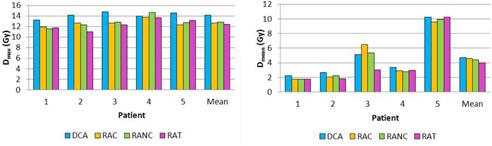
Comparison of doses to OAR ($D_{\text{max}}$ and $D_{\text{mean}}$) for DCA, ${\text{RA}}_{C},\;{\text{RA}}_{\text{NC}}$, and ${\text{RA}}_{T}$ in the case of single lesion close to an OAR.

### A. Case 1: Single lesion

The VMAT techniques improved the conformity index with 0.84±0.04 for ${\text{RA}}_{C}$ and ${\text{RA}}_{\text{NC}}$ and 0.85±0.04 for ${\text{RA}}_{T}$, whereas the DCA reached 0.77±0.05. The HI results were 0.17±0.05 for the ${\text{RA}}_{\text{NC}}$ technique compared with 0.27±0.05 for the DCA. However, DCA enhanced healthy brain protection with a $V_{10\text{Gy}}$ of 8.35±5.6 cc, while the best VMAT technique was ${\text{RA}}_{\text{NC}}$ and a $V_{10\text{Gy}}$ of 10.52±6.4 cc. For Patients 4 and 5, treatment plans were accepted despite an out of tolerance $V_{10\text{Gy}}$ for normal brain (13.37 and 20.89 cc, respectively) because of the large target volume (13.7 and 13.3 cc, respectively).

The DCA technique reduced the MU number with 3046±374. For comparison, the MU number was 4651±736, 5302±1285, and 5597±1014 for ${\text{RA}}_{\text{NC}},\;{\text{RA}}_{C}$, and ${\text{RA}}_{T}$, respectively.

### B. Case 2: Multiple lesions

${\text{RA}}_{T}$ provided higher conformity degrees with 0.80±0.08 compared with DCA with 0.71±0.08. HI results were equivalent for VMAT techniques with 0.16±0.03. For the DCA, HI was 0.24±0.07. The healthy brain protection was improved with DCA: $V_{10\text{Gy}}$ was of 9.26±4.57 cc whereas ${\text{RA}}_{\text{NC}}$ reached 13.5±6.64 cc. Concerning the MU numbers, ${\text{RA}}_{\text{NC}}$ was the technique delivering the least MUs with a decrease of 31%, 14%, and 13% compared with the DCA, ${\text{RA}}_{C}$, and ${\text{RA}}_{T}$ techniques.

### C. Case 3: Single lesion close to an OAR

The ${\text{RA}}_{T}$ increased the conformity and the target coverage homogeneity (CI=0.72±0.06 and HI=0.27±0.03) compared with the DCA (CI=0.56±0.13 and HI=0.53±0.20). On the other hand, the MU numbers were lower with the DCA with a MU decrease of 44%, 34%, and 40% compared with the ${\text{RA}}_{C},\;{\text{RA}}_{\text{NC}}$, and ${\text{RA}}_{T}$ techniques, respectively. Concerning doses to OAR, $D_{\text{max}}$ was reduced with the ${\text{RA}}_{T}$ with 12.4±1.05 Gy. For the DCA, the $D_{\text{max}}$ was 14.12±0.59 Gy. The $D_{\text{mean}}$ was slightly similar for all techniques.

## IV. DISCUSSION

In this study, the algorithms used for the DCA (PBC with iPlan RT Dose) and VMAT (AAA with Eclipse) were different. However, they were specifically commissioned to perform cranial stereotactic treatment. Because the same characteristics (dose grid step, heterogeneity correction) were used, the variances were not significant between the two algorithms for cranial treatment.

All VMAT plans, in particular the ${\text{RA}}_{T}$ were clearly superior in terms of target conformity and homogeneity compared with the DCA. This was probably due to the use of an inverse optimization algorithm which adjusted the dose to the target. Lagerwaard et al.^(27)^ compared the VMAT plan with a single dynamic conformal arc with five noncoplanar arcs for the treatment of vestibular schwannomas. They also concluded that VMAT plans consistently achieved a higher CI and decreased in areas of low‐dose irradiation. According to Wolff et al.,^(22)^ VMAT even provided a new alternative for single‐fraction SRS irradiation. Also, Gevaert et al.^(28)^ investigated the dosimetric performances of Novalis‐Tx (with dynamic conformal arcs), CyberKnife and GammaKnife for 15 patients with arterious malformation and acoustic neuromas. For DCA, our results were consistent with the Gevaert study: Paddick conformity and homogeneity indexes were, respectively, 0.66±0.03 Gy and 0.30±0.03 Gy (0.68 and 0.34 in our global study). For GammaKnife and CyberKnife, CI were similar to our VMAT techniques (CI = 0.77). Floriano et al.^(29)^ reported a mean result slightly better (CI=0.80±0.06 Gy) with 40 patients treated with CyberKnife. Massager et al.^(30)^ also investigated this parameter with 203 patients treated for a vestibular schwannomas by GammaKnife and obtained 0.77±0.08 Gy, too.

Nevertheless, the DCA enhanced healthy brain protection in all cases. Several studies exposed the link between irradiated healthy brain volume and radionecrosis development. Minniti et al.^(25)^ assessed survival and toxicity in 206 patients treated with SRS and showed that, for healthy brain whose $V_{10\text{Gy}}>12.6\;{\text{cm}}^{3}$ and $V_{12\text{Gy}}>10.9\;{\text{cm}}^{3}$, the risk of radionecrosis was 47%. They concluded for lesions $>8.5\;{\text{cm}}^{3}$, hypofractionated stereotactic radiotherapy should be considered. For Blonigen et al.,^(31)^ this proposition was also considered in case of patients with $V_{10\text{Gy}}>10.5\;{\text{cm}}^{3}$ or $V_{12\text{Gy}}>7.9\;{\text{cm}}^{3}$ to minimize the radionecrosis risk.

For patients with multiple lesions, Clark et al.^(32)^ evaluated the feasibility of single‐isocenter versus multi‐isocenter VMAT for multiple intracranial targets. They compared single arc/single isocenter (SASI) with triple arc/single isocenter (TASI) and with triple arc/triple isocenter (TATI), using dosimetric parameters. Single‐isocenter VMAT delivered a conformity equivalent to that of the multiple isocenter VMAT. The Paddick conformity index was, respectively, 0.761 (SASI), 0.699 (TASI), and 0.713 (TATI). Our results are consistent with the Clark study because the mean CI reported was of 0.77 for single‐isocenter VMAT techniques. Lee et al.,^(33)^ who compared single isocenter VMAT with the dynamic arc and IMRT plans for multiple cranial tumors, provided slightly better results for single‐isocenter VMAT, with a Paddick CI of 0.83.

On the other hand, $V_{10\text{Gy}}$ for healthy brain for VMAT techniques was higher than that of the DCA and generally out of tolerance (12.5 cc). In the literature, the tolerances for healthy brain were relative to a single lesion^25^, ^31^ and were not appropriate in this case. Instead of comparing global $V_{10\text{Gy}}$, a per‐lesion average should have been calculated, as was done in the Clark study.^(30)^

Concerning cases with a single lesion close to OAR, ${\text{RA}}_{T}$ better spared the surrounding organ at risk while conserving a high degree of conformity. Similar results were reported by Wolff et al.^(22)^ for intracranial targets and by Lagerwaard et al.^(27)^ in schwannoma vestibular cases. These two studies compared VMAT treatment with conformal arc therapy and concluded that there was a higher conformity with similar sparing of the OAR. An analogous conclusion was drawn by Fogliata et al.^(34)^ in a dosimetric study comparing IMRT, VMAT, and helical tomotherapy for benign intracranial tumors. Abacioglu et al.^(35)^ enhanced conformity index with VMAT and FFF beams and GammaKnife for vestibular schwannoma and cavernous sinus meningioma with bigger volume (respectively, 4.2 and 7.9 cc). They obtained a Paddick CI of 0.84 for GammaKnife and of 0.86 for VMAT.

Treatment time is also a parameter to be seriously taken into account because radiosurgery remains a time‐consuming technique. In the case of a single lesion (close or not to OAR), DCA delivered less MU compared with the VMAT irradiations. However, the mean overall time is much longer than ${\text{RA}}_{C}$ or ${\text{RA}}_{T}$ because of the number of couch rotations (3–4 for DCA vs. 0 and 2 for ${\text{RA}}_{C}$ and ${\text{RA}}_{T}$, respectively). Indeed, therapists had to go into the room for each arc to rotate the couch and the gantry. Consequently, the treatment time was multiplied tenfold. In addition, the more the treatment time, the more the risk intrafractional motion increases, and can become critical in the case of lesion close to OAR. On the issue of the shorter treatment delivery times, Lagerwaard et al.^(27)^ replaced conventional five‐arc radiosurgery by ${\text{RA}}_{C}$ for vestibular schwannomas. This reasoning also applies for the case of multiple lesions. ${\text{RA}}_{\text{NC}}$ delivered least MU but had more couch rotations than ${\text{RA}}_{T}$ or ${\text{RA}}_{C}$.

## V. CONCLUSIONS

This study was based on dosimetric indices and did not include the other many aspects of stereotactic treatment, such as the imaging process. For a single lesion, DCA provided better plans considering low doses to healthy brain even if quality indexes were better for the VMAT techniques. In the case of multiple lesions, VMAT plans were more appropriate than the DCA due to the conformity and homogeneity of the dose distribution and the reduction of treatment time (few couch rotation and a single isocenter). Among VMAT techniques, the ${\text{RA}}_{\text{NC}}$ were the best compromise between treatment time and healthy brain protection. Finally, the ${\text{RA}}_{T}$ performed the highest degree of quality treatment in case of the single lesion close to an organ at risk.
